# Cutaneous sequelae of a national health crisis: Obesity and the skin

**DOI:** 10.1002/ski2.7

**Published:** 2020-11-04

**Authors:** C. Cotter, S. Walsh

**Affiliations:** ^1^ Department of Dermatology King's College Hospital NHS Foundation Trust London UK

## Abstract

**Background:**

The global obesity pandemic has far‐reaching health consequences and has become a major global health challenge. The worldwide prevalence of obesity nearly doubled between 1980 and 2008 and based on the latest estimates in the European Union, obesity affects up to 30% of adults. As a consequence of this rising prevalence of obesity, there has been an increase in the frequency of certain disease of the skin.

**Objectives:**

We review the cutaneous sequelae of obesity, firstly describing the physiological consequences of increased adiposity in the skin and secondly examining the dermatoses associated with obesity.

## 1 INTRODUCTION

Obesity is defined as having a body mass index (BMI) of 30 or above.^1^, ^2^ The worldwide prevalence of obesity nearly doubled between 1980 and 2008, and in 2019, one in four adults and one in five children were obese in the United Kingdom.^3^ Onset of obesity can occur at any age and is influenced by medical, environmental, behavioural and socioeconomic circumstances. Genetic studies have implicated over 97 BMI‐associated loci.^4^ Intrauterine exposure to maternal smoke and gestational diabetes also pose an increased risk.^5^, ^6^

Adult obesity reduces longevity and its increasing prevalence has resulted in a decline in life expectancy in the 21st century.^7^ Having an elevated BMI is associated with an increased all‐cause mortality; coronary heart disease, stroke, type two diabetes, chronic kidney disease and cancer in particular.^8^ Increasing prevalence in obesity has also led to an increase in certain disease of the skin. The dermatological consequences of obesity are due to two major factors: first, the mechanical and physiological effects of an increased adipose tissue volume; second, the release of endocrine, metabolic and inflammatory peptides from distended adipocytes, acting in effect as an accessory endocrine organ.^9^

### 1.1 Physiological consequences

Obesity is associated with a wide number of changes in skin physiology. The mechanical ‘strength’ of the skin is relatively speaking reduced, due to reduced collagen deposition in relation to skin surface area.^10^ Increased water permeability of the skin leads to drier skin.^11^ Adipose tissue is a highly effective insulator and larger amounts of subcutaneous tissue impair temperature regulation.

**FIGURE 1:**
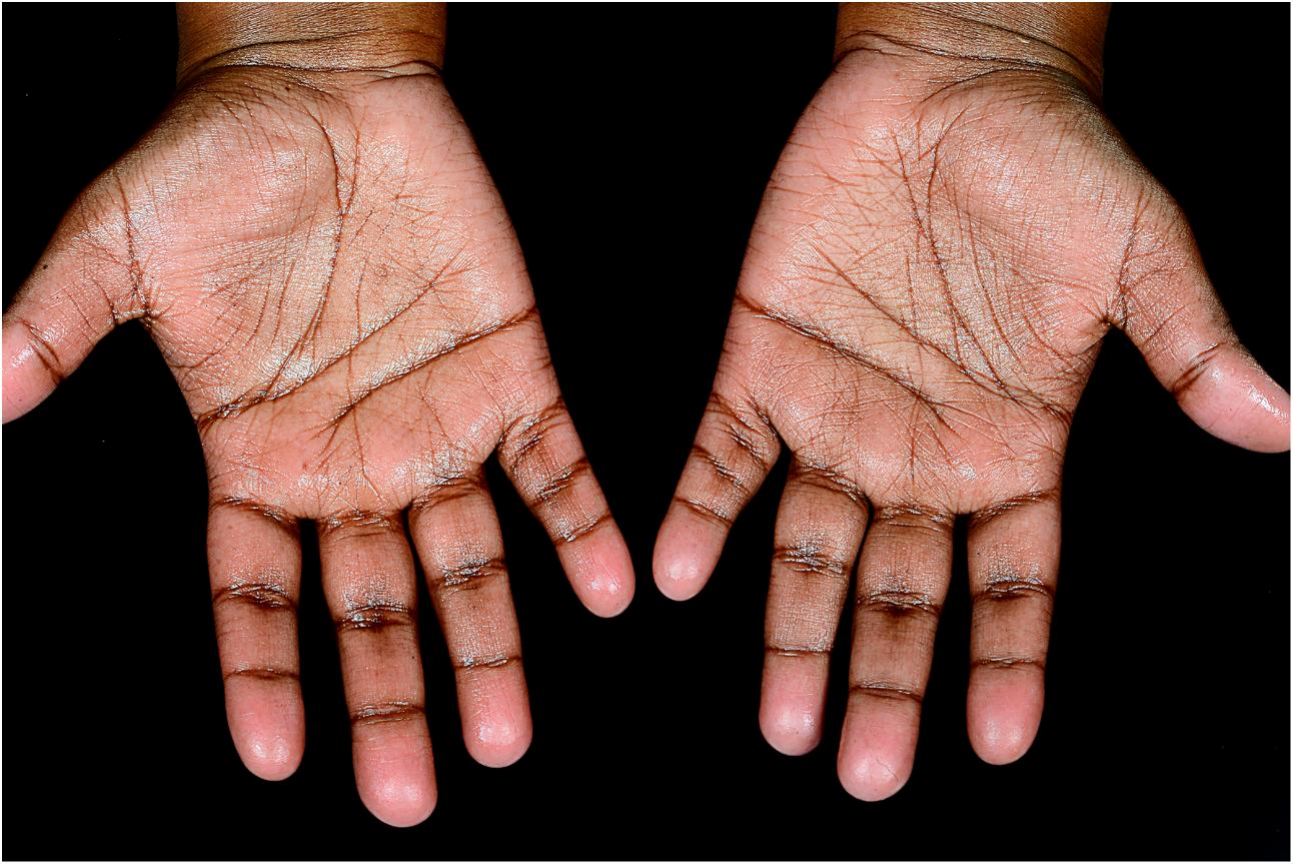
Palmar hyperhidrosis

An increase in sweat gland activity—possibly related to this impaired temperature regulation—is well recognized and there is an established ‘dose‐dependent’ association between hyperhidrosis and obesity (Figure 1).^12^ A cross‐sectional study of Israeli adolescents confirmed an incremental increase in the prevalence of hyperhidrosis from underweight to obese adolescents.^13^ Each BMI unit was correlated with an adjusted odds ratio for hyperhidrosis of 3.2% for males and 1.5% for females.

Adipose tissue is the largest organ in the body. Understanding of its endocrine nature has expanded significantly over the last 20 years.^14^ Proteomics indicate that adipose tissue can secrete more than 700 proteins.^14^ The proteins termed adipokines are secreted from both adipocytes and cells within the adipose tissue. These adipokines have diverse and extensive effects on glucose metabolism and inflammation and dysregulated adipokines are involved in the development of obesity‐related cutaneous disease^15^, ^16^

### 1.2 The microbiome and obesity

The skin is inhabited by diverse communities of bacteria, fungi and viruses which together constitute the skin microbiome.^17^ The microbiome is dynamic and is constantly adapting in response to environmental factors including diet and antimicrobial therapy. Brandwein et al demonstrated a statistical correlation between an individual's BMI and the skin microbiome.^18^ Overweight individuals exhibit a less diverse microbiome with a relative increase in corynebacterium. Corynebacterium promotes cutaneous inflammation via IL‐23‐dependent responses mediated by expression of mycolic acid in obesity and thus may be responsible for promoting disease states.^19^

The gut microbiome composition also varies in obese individuals. The Firmicutes to Bacteroidetes ratio is increased in overweight states and decreases during periods of weight loss.^20^ Conversely, calories absorbed and response to calorie deficit is influenced by the microbiome with patients who have a greater Prevotella to Bacteroides ratio losing more weight on a diminished calorie diet.^21^

### 1.3 Common skin diseases in obesity

Several dermatoses are associated with increasing BMI, the incidence of which is influenced by the degree of obesity. Striae distensae, identified as linear striation of the skin, appear due to increased mechanical stress from adipose tissue and elevated serum adrenocorticosteroids.^22^ Plantar hyperkeratosis results from friction and increased pressure related to excess body weight.^10^, ^23^

Acanthosis nigricans, recognized as velvety hyper‐pigmentation of the flexures, is a reliable indicator of hyperinsulinemia in obese individuals.^24^ Although the pathogenesis is not completely understood, its association with disorders of insulin resistance supports the role of hyperinsulinemia. One hypothesis suggests elevated insulin levels lead to stimulation of insulin‐like growth factor, activating keratinocyte and dermal fibroblast proliferation.^25^ Similarly, observational studies suggest acrochordons (skin tags) may signify underlying insulin resistance in obese patients.^26^

Intertrigo is dermatitis occurring between juxtaposed surfaces of skin.^27^ Sweating and maceration within body folds leads to irritation and overgrowth of resident microorganisms. Often overweight patients have several dermatoses superimposed upon one another.^28^ Obese patients are predisposed to irritant intertrigo and secondary infection with candida, tinea or bacteria (*Staphylococcus aureus* or Corynebacterium) is common.

### 1.4 Hidradenitis suppurativa

**FIGURE 2:**
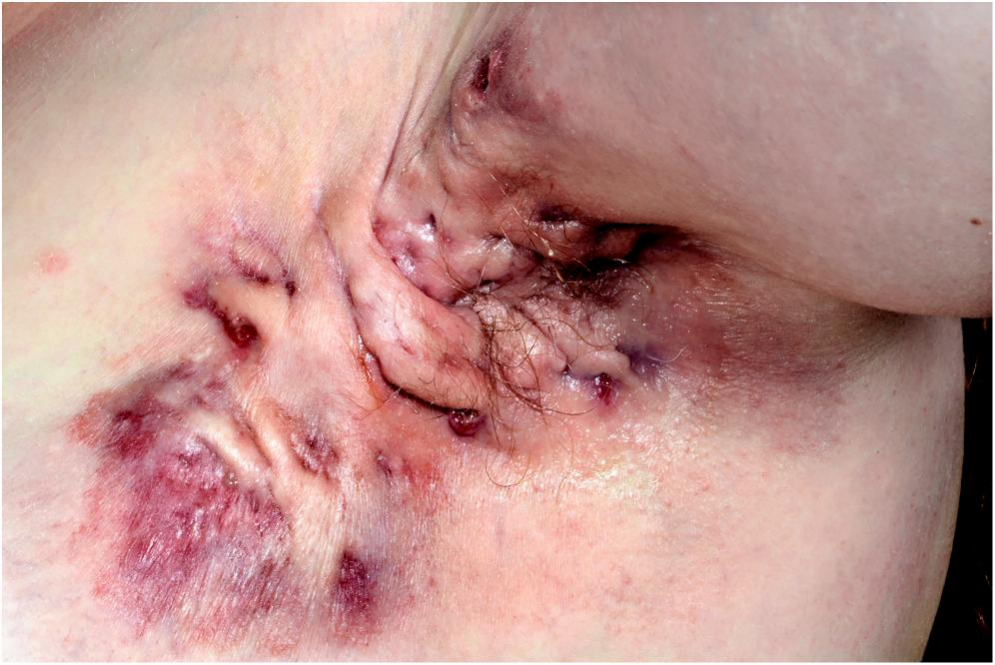
Severe hidradenitis suppurativa

Hidradenitis suppurativa (HS) is a disabling chronic inflammatory dermatosis defined by relapsing and remitting, painful abscesses, nodules and draining sinus tracts (Figure 2).^28^ Obesity is an established risk factor for HS. A study directed by Revuz et al identified an increment of the likelihood of HS diagnosis by 1.12 for each unit increase in BMI.^29^ Indeed, the point prevalence of HS in obese is almost 20% as compared to 1% of the background population.^30^ This provides compelling evidence to support the role of weight loss in reducing disease severity in HS. Weight loss results in reduced friction, changes in microbial colonization and decreased inflammation. Indeed, in a survey of patients undergoing bariatric surgery, 49% experienced disease resolution.^30^

### 1.5 Psoriasis

**FIGURE 3:**
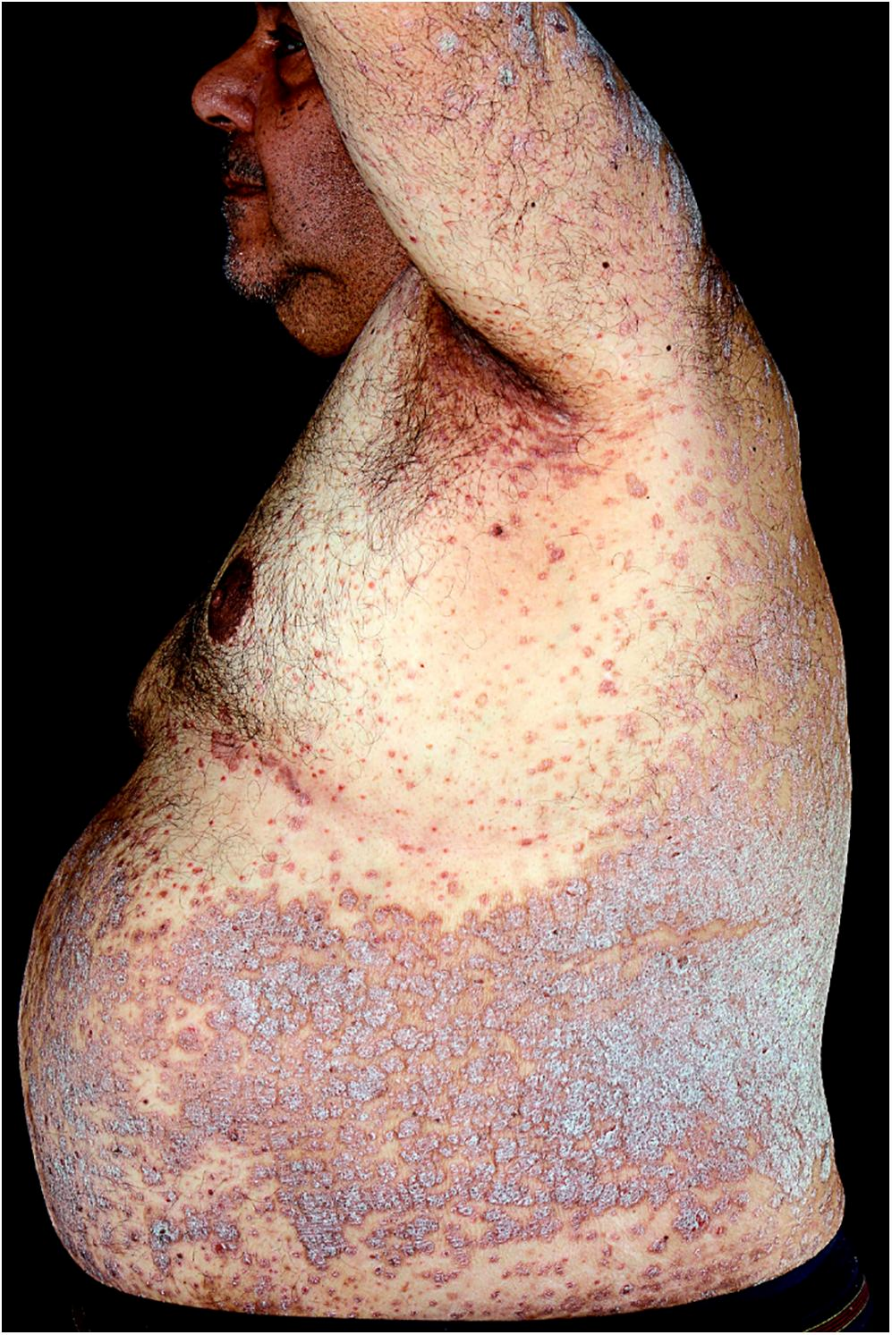
Extensive plaque psoriasis

There is an increased prevalence and incidence of obesity in patients with psoriasis and a notable dose‐dependent increase in disease severity (Figure 3).^31^, ^32^ In 10,000 psoriasis patients included in biologic therapy clinical trials, the average BMI was 30.6 kg/m^2^.^33^ TNF‐alpha, a pro‐inflammatory cytokine implicated in the pathogenesis of psoriasis, is produced by adipose tissue and found at increased levels in overweight individuals.^34^ Additional adipokines including IL‐6, leptin, resistin and adiponectin play a role.^35^ Excess body weight worsens existing psoriasis and impedes the efficacy of directed therapy, supporting the role of weight reduction in treating psoriasis.

Having a raised BMI increases both the risk of and severity chronic venous insufficiency.^36^ Obesity is also risk factor for the development of secondary lymphodema, in particular patients with morbid obesity.^37^ This is well described in breast cancer survivors with obese women having a treble‐fold increased risk of post‐operative lymphedema.^38^ Immobile morbidly obese patients are also more likely to experience pressure sores than their normal weight counterparts.^39^

### 1.6 Cellulite

**FIGURE 4:**
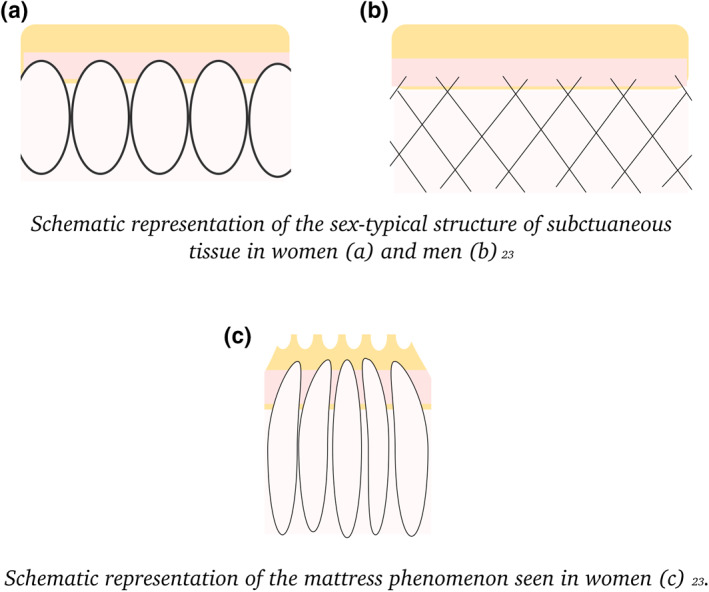
Schematic representation of the sex‐typical structure of subcutaneous tissue in (a) women and (b) men and (c) the mattress phenomenon seen in women

Cellulite is a phenomenon which has become a focus of attention for the cosmetics industry over the last 3 decades. The term derives from the French ‘*cellulite*’; however, this term translates to English as ‘cellulitis’ and has little to do with ‘cellulite’ as we understand it today. Cellulite is not a disease per se, but an appearance of the skin which develops as a consequence of the normal, sex‐typical structural characteristics of the subdermal connective tissue in women compared to men (Figure 4). Clinically recognized as orange peel skin or the ‘mattress phenomenon’, it most commonly affects skin of the buttocks, thighs and lower abdomen. It becomes more pronounced with time and may be considered a natural consequence of aging.^40^, ^41^

While the aetiology of cellulite, and the reasons why it is more prominent in some women than others, has not been fully elucidated. It appears to be due to a complex interaction between excess adiposity, the connective tissue structure of the dermis and the subcutis, and the influence of hormones on these two. There is supporting evidence for the role of hormones in theses sex‐typical subcutaneous differences by the finding of cellulite in males with androgen deficiency.^40^ Nurnberger et al identified cellulite in males with Klinefelter's syndrome with a low androgen level but not in those with a normal profile.^40^ Mattress phenomenon was also demonstrated in males following chemical castration, orchitis and following oestrogen therapy for prostate cancer.^40^ Fat loss is considered to have a role in reducing the appearance. Invasive treatments such as subcision and liposuction are available and may improve the appearance in the short term however are not without risk.^42^

### 1.7 Bariatric surgery

Bariatric surgery as a treatment for effective, sustained weight loss is becoming increasingly popular worldwide.^43^ Coined from the Greek term ‘baros’ denoting weight and ‘iatrikos’ meaning medicine, strategies of malabsorption (jejunoileal bypass and biliopancreatic diversion), restriction (gastric sleeve, gastric banding or gastroplasty) or a combination of both (roux‐en‐y) are employed.^44^ All approaches change both the anatomy and physiology of the gut leading to nutritional deficiencies.

Reduced gastric size leads to impaired grinding and release of micronutrients and limits secretion of intrinsic factor. Low levels of iron, zinc, copper, and vitamins A, E, folate and B12 may be observed with characteristic cutaneous sequelae**.** Severe protein‐energy malnutrition is seen rarely following malabsorptive procedures. Clinically described as kwashiorkor, dyschromia, superficial desquamation, sparse hair and cheilitis are seen.^45^

Bariatric surgery can also, over time, alter the anatomy and physiology of the skin. Examples would include reduction in the surface area of occluded flexural skin; a reduction in the volume of adipose tissue, leading to more efficient heat loss mechanisms and reduced sweating; changes in the skin microbiome as detailed above.

## 2 CONCLUSION

In summary, we have provided comprehensive review of the cutaneous sequelae of obesity, all which will become more prevalent as a consequence of rising obesity rates. The dermatologist should be equipped to play a role in addressing this major public health concern.

## CONFLICT OF INTERESTS

No conflict of interests have been declared.

**Table:** 

Cutaneous sequelae of obesity	
Xerosis	Increased water permeability of the skin leads to drier skin.^11^
Hyperhidrosis	Larger amounts of subcutaneous tissue impair temperature regulation.^12^
Cutaneous microbiome	Overweight individuals exhibit a less diverse microbiome with a relative increase in Corynebacterium.^19^
Striae distensae	Appear due to increased mechanical stress from adipose tissue and elevated serum adrenocorticosteroids.^22^
Plantar hyperkeratosis	Results from friction and increased pressure related to excess body weight.^10^, ^23^
Acanthosis nigricans	A reliable indicator of hyperinsulinemia in obese individuals.^24^
Acrochordons	May signify underlying insulin resistance in obese patients.^26^
Intertrigo	Often overweight patients have several dermatoses superimposed upon one another.^28^
Hidradenitis suppurativa	The point prevalence of HS in obese is almost 20% as compared to 1% of the background population.^30^
Psoriasis	Excess body weight worsens existing psoriasis and impedes the efficacy of directed therapy, supporting the role of weight reduction in treating psoriasis.^31^, ^32^
Chronic venous insufficiency	Severe chronic venous insufficiency is more likely to develop in obese individuals.^36^
Secondary lymphodema	Morbid obesity in particular is a risk factor for secondary lymphodema.^37^
Pressure sores	Immobile morbidly obese patients are more likely to experience pressure sores than their normal weight counterparts^.^ ^39^
Cellulite	Consequence of the normal, sex‐typical structural characteristics of the subdermal connective tissue in women compared to men.^40^
Bariatric surgery	Low levels of iron, zinc, copper, and vitamins A, E, folate and B12, may be observed with characteristic cutaneous sequalae**.** ^45^
